# Efficacy and safety of once-monthly Risperidone ISM^®^ in schizophrenic patients with an acute exacerbation

**DOI:** 10.1038/s41537-020-00127-y

**Published:** 2020-11-25

**Authors:** Christoph U. Correll, Robert E. Litman, Yuriy Filts, Jordi Llaudó, Dieter Naber, Ferran Torres, Javier Martínez

**Affiliations:** 1 Department of Psychiatry Research, The Zucker Hillside Hospital, Glen Oaks, NY USA; 2 Department of Psychiatry and Molecular Medicine, Donald and Barbara Zucker School of Medicine at Hofstra/Northwell, Hempstead, NY USA; 3 Department of Child and Adolescent Psychiatry, Charité Universitätsmedizin Berlin, Berlin, Germany; 4 CBH Health LLC, Gaithersburg, MD USA; 5 Department of Psychiatry, Georgetown University Medical School, Washington, DC USA; 6 Communal Noncommercial Enterprise of Lviv Regional Council, Lviv Regional Clinical Psychiatric Hospital, Lviv, Ukraine; 7 Medical Department, Laboratorios Farmacéuticos ROVI, S.A, Madrid, Spain; 8 Department of Psychiatry and Psychotherapy, Hamburg-Eppendorf University, Hamburg, Germany; 9 Medical Statistics core facility, Clinical Pharmacology Department, IDIBAPS-Hospital Clinic, Barcelona, Spain; 10 Biostatistics Unit, Faculty of Medicine, Universitat Autònoma de Barcelona, Barcelona, Spain

**Keywords:** Psychiatric disorders, Schizophrenia

## Abstract

To evaluate the efficacy and safety of Risperidone ISM^®^ against placebo in patients with acute exacerbation of schizophrenia. A multicenter, randomized, double-blind, placebo-controlled study was conducted between June 2017 and December 2018 (NCT03160521). Eligible patients received once-monthly intramuscular injections of Risperidone ISM^®^ (75 or 100 mg) or placebo for 12 weeks. The primary efficacy outcome was change in Positive and Negative Syndrome Scale (PANSS) total score from baseline to week 12. The key secondary efficacy outcome was change from baseline in Clinical Global Impressions-Severity of Illness scale (CGI-S) score. Altogether, 438 patients were randomized (1:1:1) and 390 included in the modified ITT efficacy set. The PANSS total score (mean difference, 95% CI) improved significantly from baseline to day 85 with Risperidone ISM^®^ 75 and 100 mg, with placebo-adjusted differences of −13.0 (95% CI, −17.3 to −8.8); (*p* < 0.0001), and −13.3 (−17.6 to −8.9); (*p* < 0.0001), respectively. Significantly improved mean changes were also obtained for CGI-S score from baseline to day 85 for both doses of Risperidone ISM^®^ compared with placebo −0.7 (−1.0 to −0.5); *p* < 0.0001, for both doses. The statistically significant improvement for both efficacy outcomes were observed as early as 8 days after first injection. The most frequently reported treatment-emergent adverse events were increased blood prolactin (7.8%), headache (7.3%), hyperprolactinemia (5%), and weight increase (4.8%). Neither new nor unexpected relevant safety information was recorded. Risperidone ISM^®^ provided rapid and progressive reduction of symptoms in patients with acutely exacerbated schizophrenia without need of oral risperidone supplementation or loading doses. Both doses were safe and well tolerated.

## Introduction

Schizophrenia remains one of the most severe mental disorders^1^. The suboptimal adherence to prescribed oral antipsychotic medication regimens is prevalent in patients with schizophrenia and has been associated with relapse and worsening of long-term functional and mental health outcomes^2–4^. Long-acting formulations of antipsychotic medications (LAIs), developed to promote treatment adherence, have helped to improve adherence and thus efficacy^5–8^. Despite currently available atypical LAIs, some unmet clinical needs remain, e.g., the use of loading doses or oral cotreatment for 2–3 weeks still is needed to achieve therapeutic drug levels when starting treatment with a LAI^9^ (except for one subcutaneous version of risperidone)^10^. Laboratorios Farmacéuticos ROVI, S.A., has developed a new injectable formulation of risperidone using the ISM^®^ technology. This technology is based on an in situ forming solid polymeric matrix system that contains risperidone. The suspension obtained after reconstitution produces an early, rapid, and sustained release of risperidone by 2 h after its administration and for 1 month^11^.

Risperidone ISM^®^ is a new long-acting injectable (LAI) intramuscular (IM) formulation of risperidone, for monthly administration without the need of using oral supplementation or loading doses, as it has been shown previously^11,12^.

The aim of this placebo-controlled study was to evaluate the short-term efficacy and safety of Risperidone ISM^®^ in patients with an acute exacerbation of schizophrenia.

## Results

### Patient flow and characteristics

**Fig. 1:**
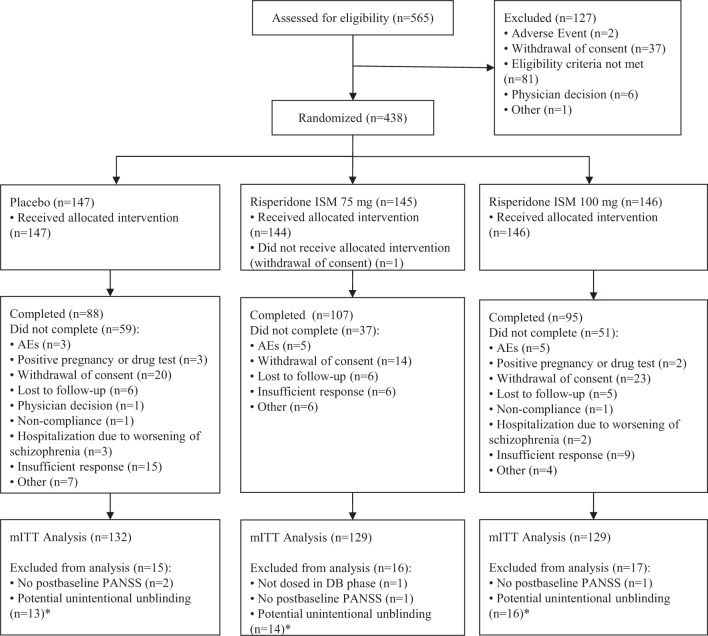
Patient disposition. Where patients marked with “*” were included in the ITT analyses (ITT: Intention-to-Treat; mITT: modified ITT).

From 565 screened patients, 438 were randomized. One of them withdrew consent before receiving the study treatment; therefore 437 were included in the safety population, and 390 counted for the modified intent-to-treat (mITT) population. The study completion was highest in the Risperidone ISM^**®**^ 75 mg group (73.8%) and lowest in the placebo group (59.9%) (Fig. 1).

Demographic and baseline characteristics were similar among treatment groups (Table 1 and Supplementary Table 1). The patients had a mean age of 42 years; 67% were men, and 48.5% were white. Most subjects were enrolled in the USA (61.1%), and the mean BMI was 28.33 kg/m^2^. The mean (SD) PANSS score at baseline was 96.4 (7.21), 96.3 (8.47), and 96.1 (8.42) for placebo, 75 and 100 mg of Risperidone ISM^**®**^, respectively.

**Table 1:** Demographic and baseline characteristics (mITT population).

Baseline variable statistic/category	Placebo *N* = 132	Risperidone ISM 75 mg *N* = 129	Risperidone ISM 100 mg *N* = 129	All Risperidone ISM *N* = 258	Overall *N* = 390
*Age (years)*					
*n*	132	129	129	258	390
Mean (SD)	40.0 (11.35)	42.6 (10.63)	42.6 (11.14)	42.6 (10.87)	41.7 (11.08)
*Sex,* *n* *(%)*					
Male	85 (64.4)	88 (68.2)	84 (65.1)	172 (66.7)	257 (65.9)
Female	47 (35.6)	41 (31.8)	45 (34.9)	86 (33.3)	133 (34.1)
*Race,* *n* *(%)*					
White	71 (53.8)	69 (53.5)	65 (50.4)	134 (51.9)	205 (52.6)
Black or African American	59 (44.7)	58 (45.0)	63 (48.8)	121 (46.9)	180 (46.2)
Asian	1 (0.8)	1 (0.8)	1 (0.8)	2 (0.8)	3 (0.8)
Other	1 (0.8)	1 (0.8)	0	1 (0.4)	2 (0.5)
*Ethnicity,* *n* *(%)*					
Hispanic or Latino	10 (7.6)	6 (4.7)	3 (2.3)	9 (3.5)	19 (4.9)
Not Hispanic or Latino	122 (92.4)	123 (95.3)	126 (97.7)	249 (96.5)	371 (95.1)
*Country,* *n* *(%)*					
Ukraine	56 (42.4)	57 (44.2)	57 (44.2)	114 (44.2)	170 (43.6)
United States	76 (57.6)	72 (55.8)	72 (55.8)	144 (55.8)	220 (56.4)
*BMI (kg/m²)*^*a*^					
*n*	132	129	129	258	390
Mean (SD)	28.19 (4.715)	27.77 (5.226)	28.26 (5.268)	28.02 (5.243)	28.07 (5.065)
*Years since Schizophrenia Diagnosis*					
*n*	132	129	129	258	390
Mean (SD)	14.3 (9.74)	16.1 (10.67)	15.7 (10.43)	15.9 (10.53)	15.4 (10.29)
*Time since Acute Exacerbation or relapse (days)*					
*n*	132	129	129	258	390
Mean (SD)	3 (1.65)	3 (1.95)	3 (3.67)	3 (2.95)	3 (2.55)

^a^BMI (kg/m^2^) is calculated as BMI = 100^2^ × weight (kg)/[Height (cm)^2^].

Note: presented statistics, frequencies and the denominator used for percentages are based on patients in the modified ITT population and the treatment received.

### Efficacy

**Fig. 2:**
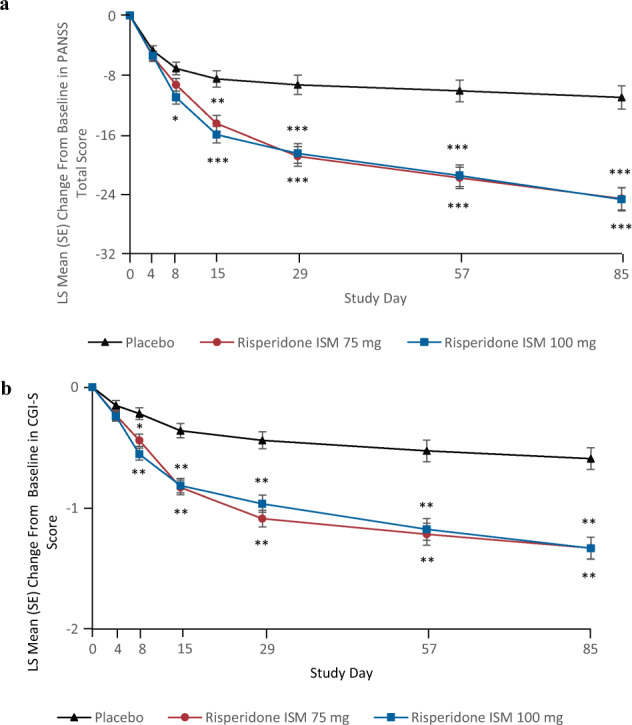
LS mean change from baseline at each time point (mITT population). **a** PANSS total score, where mean PANSS score at baseline for placebo = 96.4 (SD: 7.21), for Risperidone ISM 75 mg = 96.3 (SD: 8.47) and for Risperidone ISM 100 mg = 96.1 (SD: 8.42). The error bars represent SE and *P* values are for Risperidone ISM 75 mg and Risperidone ISM 100 mg dose group versus placebo (*p < 0.01, **p < 0.001, ***p < 0.0001), in **b** CGI-S Score, where mean CGI-S score at baseline for placebo = 4.9 (SD: 0.54), Risperidone ISM 75 mg = 4.9 (SD: 0.63) and Risperidone ISM 100 mg = 4.8 (SD: 0.53). The error bars represent SE and *P* values are for Risperidone ISM 75 mg and Risperidone ISM 100 mg dose group versus placebo (*p < 0.01, **p < 0.0001).

There was a statistically significant difference of both Risperidone ISM^**®**^ 75 mg and 100 mg versus placebo on PANSS total score mean change from baseline to Day 85. The placebo-adjusted Lawrence and Hung (LH) mean change from baseline to Day 85 was −13.0 (95% confidence interval [CI]: −17.3 to −8.8; *p* < 0.0001) and −13.3, (95% CI: −17.6 to −8.9; *p* < 0.0001) for Risperidone ISM^**®**^ 75 mg and 100 mg, respectively (Hommel adjusted *p* value < 0.0001, for both groups) (Table 2). In addition, the statistically significant improvement in PANSS total score mean change against placebo was shown as early as Day 8 for Risperidone ISM^**®**^ 100 mg (LS Mean difference, 95% CI: −3.9, −6,4 to −1.5; *p* = 0.001), and Day 15 for Risperidone ISM^**®**^ 75 mg (Fig. 2a). These significant differences remained until the end of the study (Day 85).

**Table 2:** Primary and secondary efficacy assessments at endpoint (mITT population).

Efficacy assessment	Placebo *N* = 132	Risperidone ISM 75 mg *N* = 129	Risperidone ISM 100 mg *N* = 129
*PANSS total score (mean change)*^*1*^			
Mean baseline score (SD)	96.4 (7.21)	96.3 (8.47)	96.1 (8.42)
LS mean change (SE), 95% CI^a^	−11.0 (1.56), −14.1 to −8.0	−24.6 (1.51), −27.5 to −21.6	−24.7 (1.54), −27.7 to −21.6
Treatment difference (SE), 95% CI^b^		−13.0 (2.19), −17.3 to −8.8	−13.3 (2.21), −17.6 to −8.9
* P* value^c^		<0.0001	<0.0001
*CGI-S total score (mean change)*^*2*^			
Mean baseline score (SD)	4.9 (0.52)	5.0 (0.65)	4.9 (0.48)
LS mean change (SE), 95% CI^a^	−0.6 (0.09), −0.8 to −0.4	−1.3 (0.09), −1.5 to −1.2	−1.3 (0.09), −1.5 to −1.2
Treatment difference (SE), 95% CI^b^		−0.7 (0.13), −1.0 to −0.5	−0.7 (0.13), −1.0 to −0.5
*P* value^c^		<0.0001	<0.0001
*CGI-I score*^*3*^			
LS means (SE), 95% CI	3.3 (0.10), 3.1–3.5	2.5 (0.10), 2.3–2.7	2.5 (0.10), 2.3–2.7
LS means difference (SE), 95% CI		−0.8 (0.14), −1.0 to −0.5	−0.7 (0.14), −1.0 to −0.4
*p* value		<0.0001	<0.0001
*Overall response*^*3*^			
Responders, *n* (%)	27 (20.5)	77 (59.7)	70 (54.3)
95% CI (%)	13.9–28.3	50.7–68.2	45.3–63.1
Difference in proportions (%)		39.2	33.8
95% CI		27.5 to 49.2	22.0–43.8
*P* value		<0.0001	<0.0001
*PANSS positive subscale*^*3*^			
LS Means (SE), 95% CI	−4.1 (0.50), −5.1 to −3.1	−8.0 (0.49), −9.0 to −7.1	−8.7 (0.50), −9.7 to −7.8
LS Means Difference (SE), 95% CI		−3.9 (0.70), −5.3 to −2.5	−4.6 (0.71), −6.0 to −3.2
*p* value		<0.0001	<0.0001
*PANSS negative subscale*^*3*^			
LS means (SE), 95% CI	−1.7 (0.40), −2.5 to −0.9	−3.8 (0.38), −4.5 to −3.0	−3.7 (0.39), −4.4 to −2.9
LS means difference (SE), 95% CI		−2.1 (0.55), −3.1 to −1.0	−2.0 (0.55), −3.1 to −0.9
*p* value		<0.001	<0.001
*PANSS general psychopathology subscale*^*3*^			
LS means (SE), 95% CI	−5.6 (0.82), −7.2 to −4.0	−12.8 (0.79), −14.4 to −11.3	−12.4 (0.81), −14.0 to −10.8
LS means difference (SE), 95% CI		−7.3 (1.13), −9.5 to −5.0	−6.8 (1.15), −9.1 to −4.6
* p* value		<0.0001	<0.0001

^1^Primary efficacy endpoint.

^2^Key secondary efficacy endpoint.

^3^Secondary efficacy endpoint.

*CI* confidence interval, *CGI-I* Clinical Global Impression-Improvement Scale, *CGI-S* Clinical Global Impression-Severity of Illness Scale, *ITT* Intent-to-Treat, *KM* Kaplan–Meier, *PANSS* Positive And Negative Syndrome Scale.

^a^Data were analyzed using a mixed model repeated measures (MMRM) approach.

^b^Difference (Risperidone ISM minus placebo) in least squares mean change from baseline adjusted by Lawrence and Hung method.

^C^Hommel adjusted *p* value.

For patients with baseline PANSS score ≥95, the placebo-adjusted least square mean (LSM) difference in change from baseline to Day 85 was −13.8 (95% CI: −19.5 to −8.1) and −15.6 (95% CI: −21.4 to −9.9) for Risperidone ISM^**®**^ 75 mg and 100 mg group, respectively (*p* < 0.0001 for both groups).

**Fig. 3:**
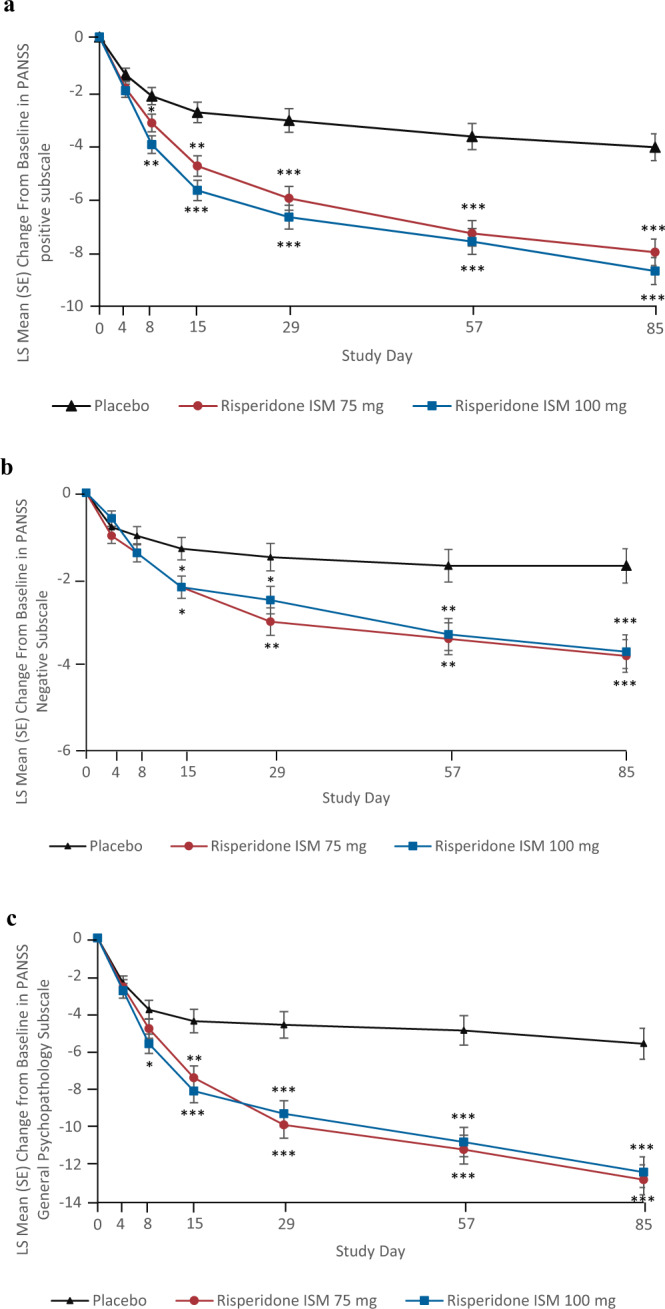
LS mean change from baseline in PANSS subscale score (mITT population). **a** PANSS positive subscale, where mean PANSS positive subscale score at baseline for placebo = 25.3 (SD: 3.11), for Risperidone ISM 75 mg = 25.1 (SD: 3.15) and for Risperidone ISM 100 mg = 25.5 (SD: 3.40). The error bars represent SE and *P* values are for Risperidone ISM 75 mg and Risperidone ISM 100 mg dose group versus placebo (*p < 0.05, **p < 0.001, ***p < 0.0001). **b** PANSS Negative Subscale, where mean PANSS score at baseline for placebo = 23.5 (SD: 3.34), for Risperidone ISM 75 mg = 23.3 (SD: 4.19) and for Risperidone ISM 100 mg = 23.1 (SD: 3.73). The error bars represent SE and *P* values are for Risperidone ISM 75 mg and Risperidone ISM 100 mg dose group versus placebo (*p < 0.05, **p < 0.01, ***p < 0.001). **c** PANSS General Psychopathology Subscale, where Mean PANSS Score at baseline: for placebo = 47.7 (SD: 4.90), for Risperidone ISM 75 mg = 47.8 (SD: 5.48) and for Risperidone ISM 100 mg = 47.4 (SD: 5.06). The error bars represent SE and P values are for Risperidone ISM 75 mg and Risperidone ISM 100 mg dose group versus placebo (*p < 0.05, **p < 0.001, *** *p* < 0.0001).

Significant differences versus placebo in mean change from baseline were observed at each assessment time point since Day 8 for PANSS Positive subscale, and Day 15 for PANSS Negative in both Risperidone ISM^**®**^ groups, and since Day 8 for General Psychopathology subscales in Risperidone ISM^**®**^ 100 mg (Fig. 3).

The placebo-adjusted CGI-S score LSM difference in change from baseline at endpoint was: −0.7 (95% CI: −1.0 to −0.5; *p* < 0.0001) for 75 mg and −0.7 (95% CI: −1.0 to −0.5; *p* < 0.0001), for 100 mg group (Hommel adjusted *p* value < 0.0001, for both groups) (Table 2). LSM change from baseline at all assessments since Day 8 was significantly greater with both doses of Risperidone ISM^**®**^ compared with placebo (Fig. 2b).

For CGI-I score, the placebo-adjusted LSM difference at Day 85 was −0.8 (95% CI: −1.0 to −0.5) for Risperidone ISM^**®**^ 75 mg, and −0.7 (95% CI: −1.0 to −0.4) for Risperidone ISM^**®**^ 100 mg (*p* < 0.0001 for both groups) (Table 2). These differences from placebo were significant at each assessment point from Day 8 and beyond for both Risperidone ISM^**®**^ doses.

**Fig. 4:**
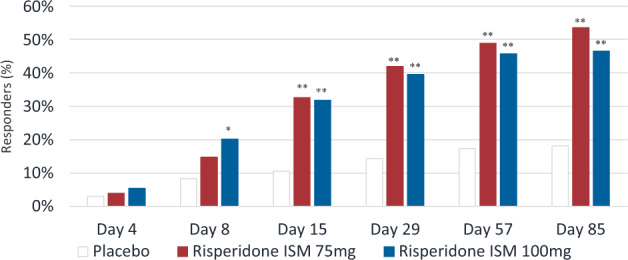
Overall response rate at each time point (mITT Population). Where the responders are those patients who achieved a decrease from baseline in PANSS Total Score >30% or CGI-I at least much improved. Dropouts prior to a presented time point are treated as non-responders and those patients who do not achieve a response are censored on the day of withdrawal/completion from the treatment. Presented statistics, frequencies, and denominator used for percentages are based on all patients in the ITT population and the randomized treatment. *p < 0.01, **p < 0.0001.

For overall response rate at endpoint, the difference in proportions versus placebo was 39.2% (95% CI: 27.5–49.2) for Risperidone ISM^**®**^ 75 mg and 33.8% (95% CI: 22.0–43.8) for Risperidone ISM^**®**^ 100 mg (*p* < 0.0001 for both groups, Mantel–Haenzel Test) (Table 2) translating into a number-needed-to-treat of three for both doses. The first significant difference from placebo in overall response rate was at day 8 for Risperidone ISM^**®**^ 100 mg (difference in proportion = 11.8%; 95% CI: 3.3–20.5; *p* = 0.005, Mantel–Haenzel test) and at Day 15 for Risperidone ISM^**®**^ 75 mg (difference in proportion = 22.0%; 95% CI: 12.1–31.5; *p* < 0.0001, Mante–Haenzel test) (Fig. 4).

Similar results for the same efficacy outcomes were obtained when the full analysis set was evaluated (Supplementary Table 2).

### Safety and tolerability

Overall, 239 (54.7%) patients experienced at least one Treatment-Emergent Adverse Event (TEAE) (Supplementary Table 3), most of them were mild (67.8%) or moderate (28.0%). Of the TEAEs, 169 (38.7%) were considered related with the study treatment. The most frequently reported drug-related TEAEs in ≥2% of patients on placebo were dizziness and headache, whereas in both Risperidone ISM^**®**^ groups (75 and 100 mg) were blood prolactin increase, hyperprolactinaemia, akathisia, and headache (Table 3).

**Table 3:** Summary of treatment-related TEAEs (safety population).

Preferred term	Placebo *N* = 147	Risperidone ISM 75 mg *N* = 144	Risperidone ISM 100 mg *N* = 146
	*n* (%)	*n* (%)	*n* (%)
Patients with at least one TEAE	32 (21.8)	60 (41.7)	77 (52.7)
Hyperprolactinaemia^a^	1 (0.7)	8 (5.6)	13 (8.9)
Injection site pain	3 (2.0)	5 (3.5)	4 (2.7)
Alanine aminotransferase increased	1 (0.7)	2 (1.4)	4 (2.7)
Blood prolactin increased^a^	0 (0)	13 (9.0)	21 (14.4)
Weight increased	2 (1.4)	5 (3.5)	6 (4.1)
Akathisia	2 (1.4)	5 (3.5)	11 (7.5)
Dizziness	4 (2.7)	4 (2.8)	5 (3.4)
Dystonia	1 (0.7)	4 (2.8)	3 (2.1)
Headache	4 (2.7)	9 (6.3)	5 (3.4)
Somnolence	2 (1.4)	4 (2.8)	8 (5.5)
Insomnia	1 (0.7)	3 (2.1)	4 (2.7)

*TEAEs* treatment-emergent adverse event, descriptions of TEAEs are coded using MedDRA version 20.0; treatment-related TEAEs listed occurred in ≥2% of risperidone ISM patients.

^a^An increase in prolactin plasma levels were considered as an adverse event (either for hyperprolactinaemia or blood prolactin increased) when any of the following criteria were present: Values above 1000 mIU/L for three consecutive determinations after randomization, although no clinical symptoms are present or Values above 530 mIU/L if clinical symptoms of hyperprolactinaemia are present (e.g., headache, decreased libido, oligo-amenorrhea).

No patient died during the study. Serious TEAEs were reported in 12 patients: 5 (3.4%), 2 (1.4%), and 5 (3.4%) with placebo, 75 and 100 mg of Risperidone ISM^**®**^ groups, respectively. Only one serious TEAE (agitation) was related to study drug in the Risperidone ISM^**®**^ 100 mg group. The incidence of patients who discontinued due to TEAE was 7.5% with placebo, 4.2% with Risperidone 75 mg and 6.2% in Risperidone 100 mg group.

Among treatment groups, the frequency of injection site reaction (ISR: redness, swelling, or induration) ranged from 6.1% with placebo, to 8.3% with Risperidone ISM^**®**^ 75 mg and 9.6% with Risperidone ISM^**®**^ 100 mg. Overall, the most frequently reported ISR was redness (6.2%), followed by swelling (1.8%) (Table 4).

**Table 4:** Evaluation of injection site reactions (safety population).

		Risperidone ISM	Risperidone ISM
Time point	Placebo	75 mg	100 mg
Statistic	*N* = 147	*N* = 144	*N* = 146
Any reaction, *n* (%)	9 (6.1)	12 (8.3)	14 (9.6)
Redness, *n* (%)	7 (4.8)	9 (6.3)	11 (7.5)
Swelling, *n* (%)	3 (2.0)	3 (2.1)	2 (1.4)
Induration, *n* (%)	0 (0)	2 (1.4)	1 (0.7)

Patients reported median Visual Analog Scale (VAS) scores (0–10), indicating injection site pain were 2.0 in all study groups (Table 5).

**Table 5:** Injection site pain (safety population).

		Risperidone ISM	Risperidone ISM
Time point	Placebo	75 mg	100 mg
Statistic	*N* = 147	*N* = 144	*N* = 146
VAS pain score			
Mean (SD)	2.4 (2.56)	2.3 (2.31)	2.5 (2.38)
95% CI	1.9 to 2.8	1.9 to 2.7	2.1 to 2.9
Median	2.0	2.0	2.0
IQR	0.0 to 4.0	0.0 to 3.0	0.0 to 4.0
Min	0	0	0
Max	10	9	10

*CI* confidence interval, *IQR* interquartile range, SD standard deviation, VAS visual analog scale. The overall summary presents incidence for each reaction category and maximum VAS score.

For hematology or biochemistry parameters, there were no notable differences between treatment arms from baseline through end of treatment and no notable changes in either treatment arm, except for prolactin. Prolactin values decreased in placebo group from baseline to end of treatment, whereas they increased in both Risperidone ISM^**®**^ groups, with mean (SD) endpoint prolactin levels being 220.6 (257.4) mIU/L with placebo, 875.4 (1080.7) mIU/L with Risperidone ISM^**®**^ 75 mg and 904.8 (810.6) mIU/L with Risperidone ISM^**®**^ 100 mg.

No noteworthy differences between treatment groups were documented for Columbia-Suicide Severity Rating Scale (C-SSRS). Two patients (1.4%) in each treatment group were documented to have had treatment-emergent suicidal behavior or ideation. Suicidal behavior or ideation worsened from baseline in two patients (1.4%) in the placebo group and one patient each (0.7%) in the Risperidone ISM^**®**^ 75 mg and 100 mg groups. No suicide attempts, aborted, or interrupted attempts were reported in any patient of any treatment group during the study.

The Abnormal Involuntary Movement Scale (AIMS), Barnes Akathisia Rating Scale (BARS), and Simpson-Angus Scale (SAS) were used to assess extrapyramidal symptoms. Regarding all three scales, treatment groups were comparable and no relevant changes from baseline to end of treatment were observed in any treatment group, i.e., mean scores (SD) for AIMS: 0.1 (1.36) for placebo, −0.1 (1.98) for Risperidone ISM 75 mg and −0.1 (1.22) for Risperidone ISM 100 mg; for BARS: 0.1 (0.98) for placebo, 0.0 (0.59) for Risperidone ISM 75 mg and 0.1 (0.87) for Risperidone ISM 100 mg, and for SAS: Mean (SD) 0.145 (2.5) for placebo, 0.085 (0.7) or Risperidone ISM 75 mg and 0.048 (1.2) for Risperidone ISM 100 mg.

Almost all patients (95.9%) took medication prior to the first dose of randomized treatment. The most common pharmacological groups of pre-baseline medications were antipsychotics (88.3%) and anxiolytics (27.5%). The most common medications were risperidone (59.3%), lorazepam (23.1%), quetiapine fumarate (16.9%), tropicamide (16.0%), and proxymetacaine hydrochloride (13.3%). Medications taken prior to the first dose of the randomized treatment were similar across treatment groups. No differences were noted in efficacy between patients who received different prior treatments.

Concomitant medications were taken by 56.5% of all patients during the study. The most common pharmacological groups were anxiolytics (37.8%), anti-inflammatory and antirheumatic products, non-steroidal anti-inflammatory agents (14.9%), and other analgesics and antipyretics (10.3%). The most common concomitant medications were lorazepam (34.6%), ibuprofen (13.7%), paracetamol (9.2%), and lisinopril (8.0%). There were no clinically relevant differences among the study groups.

## Discussion

The data obtained in the present study demonstrate the efficacy, safety, and tolerability of Risperidone ISM^**®**^ in the monthly treatment of the acute schizophrenia. Superiority of active treatment versus placebo was shown for the primary efficacy outcome, with a statistically significant advantage of both Risperidone ISM^**®**^ 75 mg and 100 mg to placebo on PANSS total score mean change from baseline to Day 85 in the mITT population,. The key secondary efficacy variable, CGI-S score mean change from baseline to Day 85, was also superior to placebo for both doses of Risperidone ISM^**®**^.

Although comparisons between studies should be interpreted with caution because of the differences in patient characteristics and study methodology, the placebo-adjusted reduction of the PANSS total score with Risperidone ISM^**®**^ (−13.0 and −13.3) was higher than those obtained by other LAIs in similar acute schizophrenia studies, being approximately twice that of RBP-7000 90 mg/120 mg at 8 weeks (−6.1 and −7.2)^13^ and Paliperidone Palmitate 25 mg eq/100 mg eq/150 mg eq at 13 weeks (−5.1, −8.7, and −9.8),^14^ as well as similar to Aripiprazole once-monthly 400 mg at 10 weeks (−15.1),^15^ and Aripiprazole Lauroxil 441 mg/882 mg at 12 weeks (−10.9 and −11.9),^16^ where patients received also oral aripiprazole during 2 and 3 weeks after randomization, respectively.

The onset of significant improvement in PANNS total and CGI-S score mean change was shown as early as Day 8 for the 100 mg dose and was maintained until the end of treatment period. Thus, Risperidone ISM^**®**^ could address an unmet medical need and be used as an early antipsychotic therapy at the admission of acutely exacerbated schizophrenic patient for a rapid and effective reduction of severe or moderate psychotic symptoms.

Risperidone ISM^**®**^ 75 mg and 100 mg were also superior to placebo for improving the patients’ Positive and Negative Symptom Scores (PANSS), overall response rates, and CGI-I scores. Improvement in CGI-I scores and PANSS-positive symptom scores compared with placebo was shown as early as Day 8 and continued until Day 85.

Similarly, a significant improvement in overall response rate was also seen as early as Day 8 for Risperidone ISM^**®**^ 100 mg and for Risperidone ISM^**®**^ 75 mg at Day 15, being significantly higher in both doses of Risperidone ISM^**®**^ versus placebo, at all subsequent time points, with a high and clinically very meaningful number-needed-treat of three.

Likewise, consistent results were demonstrated in the PANSS positive, negative, and General Psychopathology subscales. For the PANSS positive and negative subscales, statistically differences in mean change were obtained as early as Day 8 and Day 15 in positive and in negative subscales, respectively, demonstrating that Risperidone ISM^**®**^ may rapidly resolve not only positive symptoms but also negative symptoms, unlike another monthly injectable Risperidone, in which changes in PANSS-negative scale scores was not significant different across the treatment and placebo groups^13^. In addition, the effect shown in the more severely ill subpopulation (≥95 PANSS total score at baseline) further demonstrate the robust efficacy of Risperidone ISM^**®**^, and particularly with the dose of 100 mg, which produced at endpoint a particularly large reduction over placebo of 15.6 points in PANSS total score. This finding provides further evidence for the potential value of Risperidone ISM^**®**^ for the treatment of the acutely exacerbated schizophrenic patient who may need to be hospitalized owing to the severity of their symptoms.

Both doses of Risperidone ISM^**®**^ were well tolerated. The adverse events (AEs) observed were those expected for oral and LAI risperidone at therapeutic doses^17^ and were consistent with that observed in previous studies with Risperidone ISM^**®**^^11,12^.

All TEAEs were mainly mild or moderate in most patients in both treatment groups. Although the frequency of TEAEs was lower with placebo than in the Risperidone ISM^**®**^ groups, the rate was similar to those reported in a similar study in acute schizophrenia,^16^ and slightly lower to those observed in previous LAI risperidone study^13^. Furthermore, both Risperidone ISM^**®**^ groups were associated with lower rate of discontinuation owing to TEAE compared with placebo, and no patient died owing to a TEAE during the study.

Generally, the incidence of serious TEAEs and of TEAEs, leading to study drug discontinuation was low and no clear differences between treatment groups were observed. Similarly, the frequency of ISRs (redness, swelling, or induration) was low overall, with redness being the most frequent in all treatment groups, and with a slight trend for a dose-dependent increase of ISRs.

No relevant differences between treatment groups were seen in the 0–10 Visual Analog Scale (VAS) score, with a median value of 2.0 in all treatment groups, which is a clinically meaningful result for a new LAI formulation. Similarly, the EPS, akathisia, dyskinesia, and suicidality safety scales also did not indicate significant differences between either dose of Risperidone ISM^**®**^ and placebo.

There were further no significant differences in laboratory measures between treatment arms from baseline through end of the study and no notable changes in either treatment arm, except for prolactin. The events related with the prolactin increase were between the more frequently reported TEAEs in this study, with a comparable incidence to that described by others^13,18^.

Several limitations need to be considered when interpreting the study results. As this was a short-term study in acute exacerbated patients, it does not address long-term maintenance treatment, but the clinical efficacy of the active compound risperidone for maintenance treatment is very well known^6,7,19,20^. Nevertheless, a long-term evaluation of Risperidone ISM^®^ is also being carried out,^21^ whose results will be published soon. Meanwhile, the data shown here indicate that Risperidone ISM^**®**^ could be a clinical option both for the acute and maintenance treatment of schizophrenia.

Patients with schizophrenia suffering a relapse need urgent attention owing to the severity of the symptomatology and future consequences if not treated immediately^22^.

In conclusion, Risperidone ISM^**®**^ is a new monthly LAI antipsychotic that provides immediate and sustained drug plasma levels without loading doses or oral supplementation. Risperidone ISM^**®**^, using monthly intramuscular doses of 75 mg or 100 mg, was well tolerated, and provided significant improvement of the symptomatology and disease severity in acutely exacerbated patients with schizophrenia. Moreover, this efficacy was observed as early as at the 8th day after first injection and was further improved up to 12 weeks, without requiring any loading dose or supplementation with oral risperidone. Thus, Risperidone ISM^**®**^ can be an effective therapeutic strategy at the admission of patients with schizophrenia suffering from an acute episode with severe or moderate psychotic symptoms.

## Methods

### Study design

This was a phase III multicenter, randomized, double-blind, placebo-controlled clinical trial (PRISMA-3), which was conducted between June 2017 and December 2018 at 26 sites in the United States and Ukraine, in accordance with the Declaration of Helsinki, and Good Clinical Practice principles outlined in the International Conference on Harmonization. The protocol, amendments, and informed consent were approved by the Ethics Committee for each site, and written informed consent was obtained from all subjects before study participation. This study was registered at ClinicalTrials.gov (identifier: NCT03160521).

The study consisted of a screening period of up to 8 days, immediately preceding the baseline day, followed by a treatment period of 12 weeks, which ended with a 2-week follow-up period. Eligible patients were randomly assigned 1:1:1 to double-blind intramuscular treatment with 75 mg or 100 mg of Risperidone ISM^**®**^ or placebo. After initial dosing at baseline, each study drug was administered intramuscularly once every 4 weeks during the 12-week treatment period.

### Patients

Eligible subjects were 18–65 years old, with a current diagnosis of schizophrenia, according to the diagnostic and Statistical Manual of Mental Disorders, Fifth Edition criteria and a body mass index between 18.5 and 40.0 kg/m^2^. Patients were currently experiencing an acute exacerbation or relapse with a total score between 80 and 120 on the PANSS,^23^ and a score ≥4 points for ≥2 of the following positive symptom items: delusions, conceptual disorganization, hallucinatory behavior, and suspiciousness/persecution. All patients had to score of ≥4 (moderately ill or worse) on the Clinical Global Impression-Severity scale (CGI-S)^24^ and had previously had a clinically significant beneficial response after treatment with an antipsychotic other than clozapine.

Patients were excluded if improvement in PANSS total score was ≥20% between the screening visit and baseline, or with active suicidality, indicated by having answered “yes” on item 4 or 5 of the C-SSRS^25^ in the most recent episode (within the past 2 months) or having answered “yes” to any of the five items (suicidal behavior) with an episode occurring within the last year. Patients were also excluded for the presence of clinically significant comorbid neuropsychiatric disorder, lifetime history of schizoaffective or bipolar disorders, or a history of any unstable medical condition or laboratory abnormality that could interfere with the conduct of the study or compromise the well-being of the patient. Women who were pregnant or breastfeeding were also excluded.

### Treatment

Risperidone ISM^®^ (Laboratorios Farmacéuticos ROVI, S.A., Madrid, Spain) was available in a kit of two syringes, one containing Risperidone ISM^®^ plus poly lactic-co-glycolic acid (PLGA) in the form of a solid powder, and the other containing dimethyl sulfoxide, the solvent required for reconstitution. Matching placebo was also available in a 2-syringes kit, with a similar appearance but containing only PLGA in the solid power syringe.

Eligible patients were randomized 1:1:1 in a double-blind fashion to Risperidone ISM^**®**^ 75 mg, Risperidone ISM^**®**^ 100 mg or placebo, injected into the gluteal or deltoid muscle every 4 weeks on days 1, 29, and 57. A unique randomization number was assigned via Interactive Web Response System (IWRS) accessed immediately after eligibility confirmation of a patient. The doses selected for this study were supported by the results obtained from previously conducted studies,^11,12^ as well as pharmacokinetic modeling^26^.

Patients who had never taken risperidone had a brief trial of oral risperidone 2 mg/day for 3 days during the screening period to ensure lack of any hypersensitivity reactions before the first dose of study drug.

### Study assessments

Efficacy was assessed with the PANSS, CGI-S, and Clinical Global Impression-Improvements (CGI-I)^24^ at each scheduled visit. The primary efficacy endpoint was the mean change in PANSS total score from baseline to end of treatment (Day 85 or last post-baseline assessment). The key secondary efficacy endpoint was CGI-S score mean change from baseline to end of treatment. Other secondary efficacy outcomes included mean CGI-I score at endpoint and each post-baseline assessment time point, overall response rate at endpoint (defined as PANSS total score ≥30% decrease from baseline to endpoint or CGI-I score of 2 (much improved) or 1 (very much improved) at endpoint), time to reach overall response, and overall response rate at each post-baseline assessment time point, among others.

Safety was evaluated by assessment of AEs, vital signs, laboratory test, electrocardiograms, physical examinations, ISRs (redness, swelling, and induration), and scales to assess injection site pain (VAS) and extrapyramidal symptoms (AIMS;^27^ BARS^28^, and SAS^29^) as well as suicidality (C-SSRS).

### Statistical analysis

A sample size of 124 patients in the mITT population in each treatment group would have 90% power to detect a difference in means of nine (standard deviation = 20, effect size = 0.45) with a 2.5% two-sided significance level for a Risperidone ISM^**®**^ group versus the placebo group. The power to show superiority of both Risperidone ISM^**®**^ doses to placebo using the above calculation would be at least 81%. Taking into account that each of the two Risperidone ISM^**®**^ groups were tested separately against the placebo group, a Bonferroni adjustment for the alfa level was performed. A common standard deviation of 20 in two-group *t* tests was assumed.

A relatively low post-randomization dropout of 5% rate was anticipated. This assumption was re-assessed at the interim analysis and used in re-estimating the total number of randomized patients required. One unblinded interim analysis was planned to re-estimate the sample size required for the final analysis of up to 558 patients (186 patients per arm) in the mITT population. This interim analysis was to be conducted when ~50% randomized patients, had either reached study day 85 or withdrawn from the study. The decision of the independent Data Monitoring Committee (DMC) was to continue the study without modifying the sample size.

The efficacy analysis was performed on the mITT population containing all randomized patients who received ≥1 dose of study drug with a baseline measurement and ≥1 post-baseline evaluation for the PANSS, and for whom blinding was not potentially compromised (owing to a one-off error in the IWRS).

A mixed effects model with repeated measurements (MMRM) approach was fitted for patients in the mITT population with country where enrolled, visit, treatment, and treatment-by-visit interaction as fixed effects, and baseline PANSS total score as covariate. This MMRM models were used to allow for an unstructured covariance pattern between visits to be fitted, and the visit was fitted in the MMRM as a categorical factor. To utilize the endpoint result (primary outcome) in the MMRM, the endpoint results from any early termination visits were assigned to the next planned protocol visit in the MMRM.

The primary efficacy analysis was supported with sensitivity analyses. All analyses used the Cui, Hung, Wang adjustment,^30^ and the Hommel’s^31^ closed-testing correction procedure to present *p* values. Confirmatory superiority of each Risperidone ISM^**®**^ dose versus placebo was established when *p* value < 0.05. As a sensitivity analysis, the data for the primary endpoint were analyzed for the mITT population using the analysis of covariance (ANCOVA) model.

Point estimates and 95% CIs were obtained using methodology suggested by Hung and Lawrence^32^. This methodology constructs a point estimate and a 95% confidence interval that are motivated by an adaptive test statistic.

The MMRM and ANCOVA models used both observed endpoint values and imputed^33^ study day 85 values performed for the mITT population. The ANCOVA models included country and baseline PANSS total score as covariates.

The secondary efficacy endpoints were analyzed using the same model as for the primary efficacy endpoint.

Safety and tolerability analyses were performed using data from the safety population, which included all patients who received ≥1 dose of study drug.

An independent DMC monitored patient recruitment, protocol compliance, reviewed safety data, and made recommendations about any existing or potential problems.

### Reporting summary

Further information on research design is available in the Nature Research Reporting Summary linked to this article.

## Supplementary information

Supplementary Tables

Reporting Summary

## Data Availability

The data that support the findings of this study are available from the corresponding author upon reasonable request.

## References

[1] Kahn RS (2015). Schizophrenia. Nat. Rev. Dis. Prim..

[2] Keith SJ, Kane JM (2003). Partial compliance and patient consequences in schizophrenia: our patients can do better. J. Clin. Psychiatry.

[3] Ascher-Svanum H (2006). Medication adherence and long-term functional outcomes in the treatment of schizophrenia in usual care. J. Clin. Psychiatry.

[4] Kane JM, Kishimoto T, Correll CU (2013). Non-adherence to medication in patients with psychotic disorders: epidemiology, contributing factors and management strategies. World Psychiatry.

[5] Kane JM (2003). Strategies for improving compliance in treatment of schizophrenia by using a long-acting formulation of an antipsychotic: clinical studies. J. Clin. Psychiatry.

[6] Kishimoto T (2018). Effectiveness of long-acting injectable vs oral antipsychotics in patients with schizophrenia: a meta-analysis of prospective and retrospective cohort studies. Schizophr. Bull..

[7] Kishimoto T, Nitta M, Borenstein M, Kane JM, Correll CU (2013). Long-acting injectable versus oral antipsychotics in schizophrenia: a systematic review and meta-analysis of mirror-image studies. J. Clin. Psychiatry.

[8] Kane JM, Kishimoto T, Correll CU (2013). Assessing the comparative effectiveness of long-acting injectable vs. oral antipsychotic medications in the prevention of relapse provides a case study in comparative effectiveness research in psychiatry. J. Clin. Epidemiol..

[9] Correll CU (2016). The use of long-acting injectable antipsychotics in schizophrenia: evaluating the evidence. J. Clin. Psychiatry.

[10] Citrome L (2018). sustained-release risperidone via subcutaneous injection: a systematic review of RBP-7000 (PERSERIS™) for the treatment of schizophrenia. Clin. Schizophr. Relat. Psychoses.

[11] Anta L (2018). A phase II study to evaluate the pharmacokinetics, safety, and tolerability of Risperidone ISM multiple intramuscular injections once every 4 weeks in patients with schizophrenia. Int. J. Clin. Psychopharmacol..

[12] Llaudó J (2016). Phase I, open-label, randomized, parallel study to evaluate the pharmacokinetics, safety, and tolerability of one intramuscular injection of Risperidone ISM at different dose strengths in patients with schizophrenia or schizoaffective disorder (PRISMA-1). Int. Clin. Psychopharmacol..

[13] Nasser A (2016). Efficacy, safety, and tolerability of RBP-7000 once-monthly risperidone for the treatment of acute schizophrenia: an 8-week, randomized, double-blind, placebo-controlled, multicenter phase 3 study. J. Clin. Psychopharmacol..

[14] Pandina G (2010). A randomized, placebo-controlled study to assess the efficacy and safety of 3 doses of paliperidone palmitate in adults with acutely exacerbated schizophrenia. J. Clin. Psychopharmacol..

[15] Kane JM (2014). Aripiprazole once-monthly in the acute treatment of schizophrenia: findings from a 12-week, randomized, double-blind, placebo-controlled study. J. Clin. Psychiatry.

[16] Meltzer H (2015). A randomized, double-blind, placebo-controlled trial of aripiprazole lauroxil in acute exacerbation of schizophrenia. J. Clin. Psychiatry.

[17] Risperdal Consta US Package Insert. Available at: https://www.accessdata.fda.gov/drugsatfda_docs/label/2010/021346_s31_s35_s38_s39lbl.pdf (2014).

[18] Huhn M (2019). Comparative efficacy and tolerability of 32 oral antipsychotics for the acute treatment of adults with multi-episode schizophrenia: a systematic review and network meta-analysis. Lancet.

[19] Kishimoto T, Hagi K, Nitta M, Kane JM, Correll CU (2019). Long-term effectiveness of oral second-generation antipsychotics in patients with schizophrenia and related disorders: a systematic review and meta-analysis of direct head-to-head comparisons. World Psychiatry.

[20] Kishimoto T (2013). Relapse prevention in schizophrenia: a systematic review and meta-analysis of second-generation antipsychotic versus first-generation antipsychotics. Mol. Psychiatry.

[21] ClinicalTrials.gov. Study to evaluate the efficacy and safety of Risperidone ISM® in patients with acute schizophrenia: open label extension (PRISMA-3_OLE). https://clinicaltrials.gov/ct2/show/NCT03870880?term=PRISMA-3+OLE&draw=2&rank=1 (2020).

[22] Correll CU, Rubio JM, Kane JM (2018). What is the risk-benefit ratio of long-term antipsychotic treatment in people with schizophrenia?. World Psychiatry.

[23] Kay S, Fiszbein A, Opler L (1987). The positive and negative syndrome scale (PANSS) for schizophrenia. Schizophr. Bull..

[24] Guy, W. The clinician global severity and impression scales. ECDEU Assessment Manual for Psycopharmacology. Superintendent of Documents, IS Government Printing Office, Publication No. 76-338. Washington, DC: US Department of Health, Education, and Welfare. 218–222 (1976).

[25] Posner K, Oquendo MA, Gould M, Stanley B, Davies M (2007). Columbia Classification Algorithm of Suicide Assessment (C-CASA): classification of suicidal events in the FDA’s pediatric suicidal risk analysis of antidepressants. Am. J. Psychiatry.

[26] Winkler J (2015). Population pharmacokinetic modelling and simulations of long-acting intramuscular Risperidone ISM. Clin. Therapeutics.

[27] Guy, W. Abnormal Involuntary Movement Scale (AIMS). ECDEU Assessment Manual for Psychopharmacology. Rockville, MD: US Department of Health, Education and Welfare, Public Health Service, Alcohol, Drug Abuse, and Mental Health Administration, National Institute of Mental Health, Psycopharmacology Research Branch, Division of Extramural Research Programs. 534–537 (1976).

[28] Barnes TR (1989). A rating scale for drug-induced akathisia. Br. J. Psychiatry.

[29] Simpson GM, Angus JW (1970). A rating scale for extrapyramidal side effects. Acta Psychiatr. Scand. Suppl..

[30] Cui L, Hung HMJ, Wang SJ (1999). Modification of sample-size in group sequential trials. Biometrics.

[31] Hommel G (1988). A stagewise rejective multiple test procedure based on a modified Bonferroni test. Biometrika.

[32] Lawrence J, Hung HM (2003). Estimation and Confidence after Adjusting the Maximum Information. Biometrical J..

[33] Ratitch, B. & O’Kelly, M. Implementation of pattern-mixture models using standard SAS/STAT procedures. *PharmaSUG*, SG04 (2011).

