# Supplementary material for: Efficacy and safety of once-monthly Risperidone ISM® in schizophrenic patients with an acute exacerbation
Source: NPJ Schizophr. 2020 Nov 25;6:37. doi: 10.1038/s41537-020-00127-y (PMC7688968; doi:10.1038/s41537-020-00127-y)
Supplement: Supplementary file 1 — Supplementary Tables [file 41537_2020_127_MOESM1_ESM.pdf]

Supplementary Table 1. Demographic and Baseline Characteristics (Safety Population)

Baseline Variable<br>Statistic/Category	Placebo<br>N=147	Risperidone ISM 75mg<br>N=144	Risperidone ISM 100mg<br>N=146	All Risperidone ISM<br>N=290	Overall<br>N=437
Age (years)					
n	147	144	146	290	437
Mean (SD)	40.5(11.18)	42.5(10.86)	42.9(10.93)	42.7(10.88)	42.0(11.02)
Sex, n (%)					
Male	98(66.7)	98(68.1)	97(66.4)	195(67.2)	293(67.0)
Female	49(33.3)	46(31.9)	49(33.6)	95(32.8)	144(33.0)
Race, n (%)					
White	72(49.0)	73(50.7)	67(45.9)	140(48.3)	212(48.5)
Black or African American	72(49.0)	68(47.2)	78(53.4)	146(50.3)	218(49.9)
Asian	1(0.7)	2(1.4)	1(0.7)	3(1.0)	4(0.9)
Other	2(1.4)	1(0.7)	0	1(0.3)	3(0.7)
Ethnicity, n (%)					
Hispanic or Latino	11(7.5)	8(5.6)	3(2.1)	11(3.8)	22(5.0)
Not Hispanic or Latino	136(92.5)	136(94.4)	143(97.9)	279(96.2)	415(95.0)
Country, n (%)					
Ukraine	56(38.1)	57(39.6)	57(39.0)	114(39.3)	170(38.9)
United States	91(61.9)	87(60.4)	89(61.0)	176(60.7)	267(61.1)
BMI (kg/m^2) *					
n	147	144	146	290	437
Mean (SD)	28.36(4.909)	28.04(5.418)	28.58(5.423)	28.31(5.418)	28.33(5.247)
Years since Schizophrenia Diagnosis					
n	147	144	146	290	437
Mean (SD)	14.6(9.49)	16.1(10.58)	15.9(10.35)	16.0(10.44)	15.5(10.15)
Time since Acute Exacerbation or relapse (days)					
n	147	144	146	290	437
Mean (SD)	3(0.21)	3(0.25)	3(0.46)	3(0.37)	3(0.33)
Abnormal Prolactin Values at baseline/n (%)	30/147 (20.4)	25/144 (17.4)	16/145 (11.0)	41/289 (14.2)	71/436 (16.3)

* BMI (kg/m^2) is calculated as BMI = 100^2 × Weight (kg)/ [Height (cm)^2].
Prolactin Reference Range: Low: 59.4 mIU/L (female) and 44.5 mIU/L (male); High: 619.0 mIU/L (female) and 375 mIU/L (male).
Note: Presented statistics, frequencies and the denominator used for percentages are based on patients in the Safety Population and the treatment received.

Supplementary Table 2. Primary and Secondary Efficacy Assessments at Endpoint (ITT Population)

Efficacy Assessment	Placebo N=132	Risperidone ISM 75mg N=129	Risperidone ISM 100mg N=129
PANSS Total score (Mean Change)[1]			
Mean baseline score (SD)	96.4 (7.21)	96.3 (8.47)	96.1 (8.42)
LS Mean Change (SE), 95%CI[a]	-11.8 (1.48), -14.7 to -8.9	-23.9 (1.44), -26.7 to -21.0	-24.5 (1.46), -27.4 to -21.6
Treatment Difference (SE), 95%CI[b]		-11.6 (2.07), -15.7 to -7.5	-12.3 (2.09), -16.4 to -8.2
P-value[c]		<0.0001	<0.0001
CGI-S Total score (Mean Change)[2]			
Mean baseline score (SD)	4.9 (0.52)	5.0 (0.65)	4.9 (0.48)
LS Mean Change (SE), 95%CI[a]	-0.6 (0.09), -0.8 to -0.4	-1.3 (0.08), -1.4 to -1.1	-1.3 (0.08), -1.4 to -1.1
Treatment Difference (SE), 95%CI[b]		-0.6 (0.12), -0.9 to -0.4	-0.7 (0.12), -0.9 to -0.4
P-value[c]		<0.0001	<0.0001
CGI-I Score[3]			
LS Means (SE), 95% CI	3.2 (0.10), 3.0 to 3.4	2.5 (0.9), 2.4 to 2.7	2.5 (0.10), 2.3 to 2.7
LS Means Difference (SE), 95%CI		-0.6 (0.14), -0.9 to -0.4	-0.6 (0.14), -0.9 to -0.4
p-value		<0.0001	<0.0001
Overall Response[3]			
Responders, n (%)	35 (24.1)	84 (58.7)	78 (53.8)
95% CI (%)	17.4 to 31.9	50.2 to 66.9	45.3 to 62.1
Difference in proportions (%)		34.6	29.7
95% CI		23.4 to 44.5	18.5 to 39.7
P-value		<0.0001	<0.0001
PANSS Positive Subscale[3]			
LS Means (SE), 95% CI	-4.3 (0.48), -5.3 to -3.4	-7.7 (0.47), -8.6 to -6.8	-8.6 (0.48), -9.5 to -7.6
LS Means Difference (SE), 95% CI		-3.4 (0.67), -4.7 to -2.1	-4.3 (0.68), -5.6 to -2.9
p-value		<0.0001	<0.0001
PANSS Negative Subscale[3]			
LS Means (SE), 95% CI	-1.9 (0.38), -2.6 to -1.1	-3.7 (0.36), -4.4 to -3.0	-3.7 (0.37), -4.5 to -3.0
LS Means Difference (SE), 95% CI		-1.8 (0.53), -2.9 to -0.8	-1.9 (0.53), -2.9 to -0.8
p-value		<0.001	<0.001
PANSS General Psychopathology Subscale[3]			
LS Means (SE), 95% CI	-6.0 (0.77), -7.5 to -4.5	-12.5 (0.75), -14.0 to -11.0	-12.4 (0.81), -14.0 to -10.8
LS Means Difference (SE), 95% CI		-6.5 (1.07), -8.6 to -4.4	-6.4 (1.08), -8.5 to -4.2
p-value		<0.0001	<0.0001

[1] Primary efficacy endpoint; [2] Key secondary efficacy endpoint; [3] Secondary efficacy endpoint; CI = Confidence Interval; CGI-I = Clinical Global Impression-Improvement Scale, CGI-S = Clinical Global Impression-Severity of Illness Scale, ITT = Intent to Treat, KM = Kaplan-Meier; PANSS = Positive and Negative Syndrome Scale. [a] Data were analyzed using a mixed model repeated measures (MMRM) approach. [b] Difference (Risperidone ISM minus placebo) in least squares mean change from baseline adjusted by Lawrence and Hung method. [C] Hommel adjusted p-value.

Supplementary Table 3. Summary of TEAEs (Safety Population)

Preferred Term	Placebo<br>N=147	Risperidone ISM<br>75 mg<br>N=144	Risperidone ISM<br>100 mg<br>N=146
	n (%)	n (%)	n (%)
Patients with at least one TEAE	65 (44.2)	80 (55.6)	94 (64.4)
Patients with related TEAEs	32 (21.8)	60 (41.7)	77 (52.7)
Patient with at least one TEAE leading to treatment discontinuation	11 (7.5)	6 (4.2)	9 (6.2)
Tachycardia	0 (0)	2 (1.4)	4 (2.7)
Hyperprolactinaemia*	1 (0.7)	8 (5.6)	13 (8.9)
Constipation	2 (1.4)	4 (2.8)	2 (1.4)
Injection site pain	5 (3.4)	8 (5.6)	4 (2.7)
Nasopharyngitis	0 (0)	5 (3.5)	4 (2.7)
Alanine aminotransferase increased	3 (2.0)	4 (2.8)	7 (4.8)
Aspartate aminotransferase increased	3 (2.0)	2 (1.4)	4 (2.7)
Blood prolactin increased*	0 (0)	13 (9.0)	21 (14.4)
Blood triglycerides increased	1 (0.7)	4 (2.8)	3 (2.1)
Weight increased	3 (2.0)	10 (6.9)	8 (5.5)
Akathisia	3 (2.0)	6 (4.2)	11 (7.5)
Dizziness	4 (2.7)	5 (3.5)	6 (4.1)
Dystonia	1 (0.7)	4 (2.8)	3 (2.1)
Headache	5 (3.4)	15 (10.4)	12 (8.2)
Somnolence	4 (2.7)	4 (2.8)	8 (5.5)
Insomnia	6 (4.1)	4 (2.8)	6 (4.1)
Schizophrenia	7 (4.8)	3 (2.1)	3 (2.1)

TEAEs = Treatment-emergent Adverse Event; Descriptions of TEAEs are coded using MedDRA version 20.0; Treatment-related TEAEs listed occurred in ≥ 2% of Risperidone ISM patients.

*An increase in prolactin plasma levels was considered as an adverse event (either for "hyperprolactinaemia" or "blood prolactin increased") when any of the following criteria were present: Values above 1000 mIU/L (=47 ng/mL) for 3 consecutive determinations after randomization, although no clinical symptoms are present, or values above 530 mIU/L (=23.5 ng/mL) if clinical symptoms of hyperprolactinaemia are present (e.g., headache, decreased libido, oligo-amenorrhea).
