# Supplementary material for: Efficacy and safety of once-monthly Risperidone ISM® in schizophrenic patients with an acute exacerbation
Source: NPJ Schizophr. 2020 Nov 25;6:37. doi: 10.1038/s41537-020-00127-y (PMC7688968; doi:10.1038/s41537-020-00127-y)
Supplement: Supplementary file 2 — Reporting Summary [file 41537_2020_127_MOESM2_ESM.pdf]

nature research

Corresponding author(s): DBPR/NPJSCHZ00510

Last updated by author(s): Sep 18, 2020

Reporting Summary

Nature Research wishes to improve the reproducibility of the work that we publish. This form provides structure for consistency and transparency in reporting. For further information on Nature Research policies, see our Editorial Policies and the Editorial Policy Checklist.

Statistics

For all statistical analyses, confirm that the following items are present in the figure legend, table legend, main text, or Methods section.

n/a	Confirmed	
☐	☒	The exact sample size (n) for each experimental group/condition, given as a discrete number and unit of measurement
☒	☐	A statement on whether measurements were taken from distinct samples or whether the same sample was measured repeatedly
☐	☒	The statistical test(s) used AND whether they are one- or two-sided<br>*Only common tests should be described solely by name; describe more complex techniques in the Methods section.*
☐	☒	A description of all covariates tested
☐	☒	A description of any assumptions or corrections, such as tests of normality and adjustment for multiple comparisons
☐	☒	A full description of the statistical parameters including central tendency (e.g. means) or other basic estimates (e.g. regression coefficient) AND variation (e.g. standard deviation) or associated estimates of uncertainty (e.g. confidence intervals)
☐	☒	For null hypothesis testing, the test statistic (e.g. F, t, r) with confidence intervals, effect sizes, degrees of freedom and P value noted<br>*Give P values as exact values whenever suitable.*
☒	☐	For Bayesian analysis, information on the choice of priors and Markov chain Monte Carlo settings
☒	☐	For hierarchical and complex designs, identification of the appropriate level for tests and full reporting of outcomes
☒	☐	Estimates of effect sizes (e.g. Cohen's d, Pearson's r), indicating how they were calculated

Our web collection on statistics for biologists contains articles on many of the points above.

Software and code

Policy information about availability of computer code

Data collection	Data were collected using Medidata Classic Rave® 2019.1.3
Data analysis	All statistical analyses were done using SAS statistical software version 9.3 or higher

For manuscripts utilizing custom algorithms or software that are central to the research but not yet described in published literature, software must be made available to editors and reviewers. We strongly encourage code deposition in a community repository (e.g. GitHub). See the Nature Research guidelines for submitting code & software for further information.

Data

Policy information about availability of data

All manuscripts must include a data availability statement. This statement should provide the following information, where applicable:

- Accession codes, unique identifiers, or web links for publicly available datasets
- A list of figures that have associated raw data
- A description of any restrictions on data availability

The data that support the findings of this study are available from the corresponding author upon reasonable request.

Field-specific reporting

Please select the one below that is the best fit for your research. If you are not sure, read the appropriate sections before making your selection.

☒ Life sciences ☐ Behavioural & social sciences ☐ Ecological, evolutionary & environmental sciences

For a reference copy of the document with all sections, see nature.com/documents/nr-reporting-summary-flat.pdf

Life sciences study design

All studies must disclose on these points even when the disclosure is negative.

Sample size	A sample size of 124 patients in the mITT population in each treatment group would have 90% power to detect a difference in means of 9 (standard deviation = 20, effect size = 0.45) with a 2.5% 2-sided significance level for a Risperidone ISM group versus the placebo group. The power to show superiority of both Risperidone ISM doses to placebo using the above calculation would be at least 81%. Taking into account that each of the two Risperidone ISM groups were tested separately against the placebo group, a Bonferroni adjustment for the alfa level was performed. A common standard deviation of 20 in 2-group t-tests was assumed.<br>A relatively low post-randomization dropout of 5% rate was anticipated. This assumption was re-assessed at the interim analysis and used in re-estimating the total number of randomized patients required. One unblinded interim analysis was planned to re-estimate the sample size required for the final analysis of up to 558 patients (186 patients per arm) in the mITT population. This interim analysis was to be conducted when approximately 50% randomized patients, had either reached study day 85 or withdrawn from the study. The decision of the independent DMC was to continue the study without modifying the sample size.
Data exclusions	One randomized patient was not included in any of the analyses because did not received study medication.
Replication	It were performed sensitivity analyses wich included:<br>- Analysis of PANSS total score mean change from baseline to endpoint, in the mITT population using observed cases (OC) only (i.e. when the last post-baseline double-blind assessment was performed). For this analysis, change from baseline at endpoint was analyzed using an Analysis of Covariance (ANCOVA) model with country where enrolled and treatment as fixed effects and baseline PANSS total score as a covariate.<br>- Analysis of the PANSS total score mean change from baseline to day 85, in the modified randomized population using an ANCOVA model with country where enrolled and treatment as fixed effects and baseline PANSS total score as a covariate.
Randomization	Eligible patients were randomized (1:1:1) to receive once-monthly intramuscular injections of Risperidone ISM® (75 or 100 mg) or placebo for 12 weeks.<br>A unique randomization number was assigned via Interactive Web Response System (IWRS) accessed immediately after eligibility confirmation of a patient. The randomization process will determine (under double-blind conditions) for each individual patient the study drug regimen: an individual patient will receive either active Risperidone ISM (75 mg or 100 mg) or placebo.
Blinding	The study is a double-blind clinical trial. The treatment assignment for each individual patient was reminded blinded for patients and investigators during data collection and analysis.

Reporting for specific materials, systems and methods

We require information from authors about some types of materials, experimental systems and methods used in many studies. Here, indicate whether each material, system or method listed is relevant to your study. If you are not sure if a list item applies to your research, read the appropriate section before selecting a response.

Materials & experimental systems

n/a	Involved in the study	
☒	☐	Antibodies
☒	☐	Eukaryotic cell lines
☒	☐	Palaeontology and archaeology
☒	☐	Animals and other organisms
☐	☒	Human research participants
☐	☒	Clinical data
☒	☐	Dual use research of concern

Methods

n/a	Involved in the study	
☒	☐	ChIP-seq
☒	☐	Flow cytometry
☒	☐	MRI-based neuroimaging

Human research participants

Policy information about studies involving human research participants

Population characteristics	Eligible subjects were 18-65 years old, with a current diagnosis of schizophrenia, according to the diagnostic and Statistical Manual of Mental Disorders, Fifth Edition (DSM-5) criteria and a body mass index between 18.5 and 40.0 kg/m2. Patients were currently experiencing an acute exacerbation or relapse with a total score between 80 and 120 on the Positive and Negative Syndrome Scale (PANSS), and a score ≥4 points for ≥2 of the following positive symptom items: delusions,

	conceptual disorganization, hallucinatory behavior and suspiciousness/persecution. All patients had to score of ≥4 (moderately ill or worse) on the Clinical Global Impression-Severity scale (CGI-S) and had previously had a clinically significant beneficial response after treatment with an antipsychotic other than clozapine. REVISAR
Recruitment	The clinical trial was conducted at 26 sites in the United States and Ukraine where participants were recruited among their settings, either inpatient or outpatients with an acute exacertabion of schizophrenia.
Ethics oversight	The protocol, amendments, and informed consent were approved by the Ethics Committee for each site.<br>For all sites from the USA, Copernicus Group Independent Review Board (CGIRB) and for the Ukraine centers were the following Ethics Committee:<br>Ethics Committee of Kyiv City<br>Ethics Committee of Communal Institution of Lviv Regional Council<br>Ethics Committee of Communal Non-profit Enterprise of Kharkiv Regional Council<br>Ethics Committee of Communal Establishment Kherson Regional Psychiatric Hospital of Kherson Regional Council<br>Ethics Committee of Communal Establishment Acad. O.I.Iushchenko Vinnytsia Regional Psychoneurological Hospital<br>Ethics Committee of Communal Enterprise Poltava Regional Clinical Psychiatric Hospital<br>Ethics Committee of Communal Non-profit Enterprise Odesa Regional Medical Centre of Mental Health<br>Ethics Committee of Kyiv Regional Medical Association<br>Ethics Committee of Communal Institution Dnipropetrovsk Regional Clinical Hospital<br>Ethics Committee of Communal Institution of Lviv Regional Council

Note that full information on the approval of the study protocol must also be provided in the manuscript.

Clinical data

Policy information about clinical studies

All manuscripts should comply with the ICMJE guidelines for publication of clinical research and a completed CONSORT checklist must be included with all submissions.

Clinical trial registration	NCT03160521
Study protocol	The main information of the study protocol can be accessed through clinicalTrials.gov database. The full study protocol is available from the corresponding author upon reasonable request.
Data collection	This clinical trial (PRISMA-3) was conducted between 06/2017 and 12/2018 at 26 sites in the United States and Ukraine
Outcomes	The Primary Outcome Measure was Positive and Negative Syndrome Scale (PANSS) total score. The primary endpoint was assessed by the PANSS total score mean change from baseline to end of treatment. The key secondary outcome measure was Clinical Global Impression-Severity scale (CGI-S) Score. It was assessed by the CGI-S score mean change from baseline to end of treatment.
